# Breast Cancer Diagnosis Using a Microfluidic Multiplexed Immunohistochemistry Platform

**DOI:** 10.1371/journal.pone.0010441

**Published:** 2010-05-03

**Authors:** Minseok S. Kim, Taemin Kim, Sun-Young Kong, Soim Kwon, Chae Yun Bae, Jaekyu Choi, Chul Hwan Kim, Eun Sook Lee, Je-Kyun Park

**Affiliations:** 1 Department of Bio and Brain Engineering, Korea Advanced Institute of Science and Technology (KAIST), Daejeon, Republic of Korea; 2 Department of Electrical Engineering and Computer Science, Korea Advanced Institute of Science and Technology (KAIST), Daejeon, Republic of Korea; 3 Department of Laboratory Medicine, Research Institute and Hospital, National Cancer Center, Goyang, Republic of Korea; 4 Department of Breast and Endocrine Surgery, College of Medicine, Korea University, Seoul, Republic of Korea; 5 Department of Pathology, College of Medicine, Korea University, Seoul, Republic of Korea; 6 KAIST Institute of NanoCentury, Daejeon, Republic of Korea; Health Canada, Canada

## Abstract

**Background:**

Biomarkers play a key role in risk assessment, assessing treatment response, and detecting recurrence and the investigation of multiple biomarkers may also prove useful in accurate prediction and prognosis of cancers. Immunohistochemistry (IHC) has been a major diagnostic tool to identify therapeutic biomarkers and to subclassify breast cancer patients. However, there is no suitable IHC platform for multiplex assay toward personalized cancer therapy. Here, we report a microfluidics-based multiplexed IHC (MMIHC) platform that significantly improves IHC performance in reduction of time and tissue consumption, quantification, consistency, sensitivity, specificity and cost-effectiveness.

**Methodology/Principal Findings:**

By creating a simple and robust interface between the device and human breast tissue samples, we not only applied conventional thin-section tissues into on-chip without any additional modification process, but also attained perfect fluid control for various solutions, without any leakage, bubble formation, or cross-contamination. Four biomarkers, estrogen receptor (ER), human epidermal growth factor receptor 2 (HER2), progesterone receptor (PR) and Ki-67, were examined simultaneously on breast cancer cells and human breast cancer tissues. The MMIHC method improved immunoreaction, reducing time and reagent consumption. Moreover, it showed the availability of semi-quantitative analysis by comparing Western blot. Concordance study proved strong consensus between conventional whole-section analysis and MMIHC (*n* = 105, lowest Kendall's coefficient of concordance, 0.90). To demonstrate the suitability of MMIHC for scarce samples, it was also applied successfully to tissues from needle biopsies.

**Conclusions/Significance:**

The microfluidic system, for the first time, was successfully applied to human clinical tissue samples and histopathological diagnosis was realized for breast cancers. Our results showing substantial agreement indicate that several cancer-related proteins can be simultaneously investigated on a single tumor section, giving clear advantages and technical advances over standard immunohistochemical method. This novel concept will enable histopathological diagnosis using numerous specific biomarkers at a time even for small-sized specimens, thus facilitating the individualization of cancer therapy.

## Introduction

Accurate prediction and prognosis are the most critical and difficult issues in breast cancer treatment. Because breast cancer, a leading cause of cancer death in women, is a heterogeneous disease that has several biological subtypes, single biomarker test is insufficient to predict the clinical outcome of individual neoplasms [1], [2]. Many potential biomarkers with clinical value have been identified through advances in genomics, proteomics, and molecular pathology [3], and they have facilitated various kinds of personalized therapy for cancer patients [4]. However, this transition toward personalized therapy will require novel analytical methodology for accurate prediction and prognosis, particularly multiplex analysis [5], [6]. For example, genomic techniques such as DNA microarray analysis (examining 4968 significant genes) [7] and RT-PCR analysis of formalin-fixed, paraffin-embedded tissues (examining 21 prospectively selected genes) [8] have been used for chemotherapy treatment planning in breast cancer. In addition, novel genetic and molecular classifications of breast cancers have assisted in the individualization of adjuvant systemic endocrine chemotherapy, and have reduced the severity of side effects [1], . However, although these genomic approaches provide valuable information for breast cancer prognosis, significant changes in gene expression may not be reflected in the level of protein expression or practical function [12]. Therefore, to obtain a more accurate and sophisticated understanding of patient status, the development of a novel analytical method to detect various biomarkers at the proteomic level is critical, in addition to analysis at the genomic level.

Immunohistochemistry (IHC) has been widely used for assessing therapeutic biomarkers and has become a major part of practical diagnosis for various malignancies in surgical pathology [13]. IHC allows the identification of proteins of interest and provides information on protein localization and tissue morphology [14]. In addition, many studies showing the relationship between immunohistochemical profiles and molecular classification of breast cancers support that IHC might play a significant role in subclassification of breast cancer patients [15], [16]. Therefore, IHC-based assays can represent an ideal method to realize personalized-tailored therapies if efficient multiplexing method is created. However, conventional IHC has been faced with several practical limitations to examine tens of biomarkers in clinics: time, labor, diagnostic expense, and the amount of tissues required. For example, when various target proteins were examined by IHC for precise prediction and prognosis such as Oncotype Dx which examines over 20 genes involved in breast cancer [17], much time and labors are required. Although an automated IHC machine is able to overcome these issues, not only high costs from many biomarkers but much tissue consumption still remain [3], which might be significantly raised as practical problems for personalized medicine. Moreover, qualitative evaluation, subjective decision and variable result in IHC represent other hurdles toward a robust and reputable proteomic tool [13].

Recently, multicolor-based IHC studies have been reported with molecular dyes and quantum dots (QDs) for multiplexing [18]−[21]. Although the multicolor IHC including direct and indirect sequential staining methods has a unique advantage of co-expressions for biomarkers, several drawbacks are accompanied depending on the multicolor staining method [22], [23]. They include low stability of primary antibodies from probe conjugation process, alteration of binding properties, difficulty of probe conjugation to antibodies, high cost of reagents, increases of time and labors, and cross-over nonspecific binding of secondary probes. Therefore, a parallel multiplexing approach is gaining the interest as an alternative to overcome the limitations of multicolor-based IHC and to enhance throughput of biomarker multiplexing. Unfortunately, to date, few studies for parallel multiplexing approach have been reported.

Here, we report a novel microfluidic parallel-multiplexed immunohistochemistry (MMIHC) platform for the quantitative pathological diagnosis of breast cancers. Since microfluidics enables the formation of a well-confined microenvironment [24], [25], with fast and easy fluidic control [26] and the precise manipulation of fluids [27]−[30], not only the variation of immunohistochemical staining, the amount of time and labor required can be reduced via automation, but also multiple biomarkers can be analyzed on a limited cancer core area. In addition, because microfluidics uses much smaller volumes of reagents and antibodies, it allows cost-effective diagnosis and reduces financial burden of patients [31]. However, most microfluidic devices have been fabricated by using an irreversible bond between a microchannel and a glass slide, and only a few studies have introduced the interface between tissue slide and a microfluidic device; this also proves that few studies applied to human clinical specimens have been reported in microfluidics. In order to apply conventional thin-section tissues into on-chip without any additional modification process, a tissue slide-compatible assembler was developed for perfect compatibility of conventional IHC method, which was robust and optimal in a microfluidic device.

The goal of this study was to demonstrate significant improvement of IHC performances in reduction of labor and tissue consumption, quantification, consistency, sensitivity, specificity, cost-effectiveness, precise diagnosis and massive multiplexing. By comparing the biomarker scores from MMIHC platform with those of conventional whole-section analysis of breast cancer tissues, the usefulness of MMIHC platform to predict patient prognosis as well as to select drugs for chemotherapy was also evaluated.

## Results

### Operation of the MMIHC Platform

**Figure 1:**
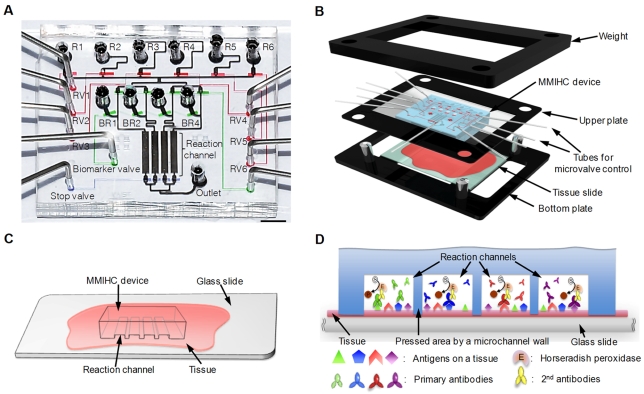
Configuration of a MMIHC platform. (**A**) Design of a MMIHC device. Reagent reservoirs (R1−R6) with individual reservoir valves (RV1−RV6), biomarker reservoirs (BR1−BR4) and four straight reaction channels were designed. After connecting a syringe pump to the outlet, the solutions required for IHC were drawn through the reaction channels in the appropriate sequence (withdrawal pumping). Scale bar, 3 mm. (**B**) Configuration of a MMIHC platform. When the microvalve controller modules were connected with the MMIHC device, the upper plate combined with the MMIHC device was aligned with the tissue. Then, a weight was mounted on the upper plate to create a reversible seal. (**C**) Schematic of the interface assembly between a MMIHC device and a tissue. (**D**) Magnified cross-sectional view of the area connecting a tissue and reaction channels of the MMIHC device. The tissue is pressed by microchannel walls dividing reaction channels, perfectly forming microchannels without any leakage. Different antibodies are flowed at each reaction channel and immunohistochemical staining for different biomarkers happened at every reaction channel, but not the area pressed by the microchannel walls.

The design of the MMIHC device took into consideration 1) the number of solutions required for IHC, 2) the number of representative biomarkers in breast cancer, and 3) the appropriate reaction channel dimensions. We selected the biomarkers with the most frequently used and the most significant indicators in therapeutic decisions of breast cancers [21], so that estrogen receptor (ER), human epidermal growth factor receptor 2 (HER2), progesterone receptor (PR), and Ki-67 were chosen and thereby four straight reaction channels were designed. Accordingly, the device was composed of six reservoirs for reagents (R1−R6), four biomarker reservoirs (BR1−BR4), individual microvalves for reservoirs of reagents and biomarkers (RV1−RV6 and biomarker valve), four reaction channels, and one outlet (Figure 1A). Because biomarkers are investigated within a localized area among whole tissue in this approach, as in tissue microarray (TMA), the dimensions of the reaction channels were considered with the human breast tumor sample size in diameter and the partial area representing a tissue result. Recent results provided by the National Cancer Center of Republic of Korea have shown that >93% of biopsy breast tissues had tumor sizes >4 mm in diameter. Therefore, each reaction channel was 800 µm in width and 5 mm in length to apply to most of tissue sections, giving an area that was 14-fold larger than that of TMA with a 600-µm diameter.

The MMIHC device was fabricated via two-step multilayer soft lithography, poly(dimethylsiloxane) (PDMS; Sylgard 184; Dow Corning, MA) replica molding and aligning processes. Creating an appropriate interface between the MMIHC device and the tissue slide was one of the most important works in realizing chip-based MMIHC and minimizing tissue damage. A weight was set on top of the device to provide constant pressure and to create a reversible seal and a robust interface; in addition, this apparatus was quick and easy to assemble as shown in Figure 1B. In the assembly process of MMIHC assay, a tissue slide was loaded onto the bottom plate. The tissue was soaked with washing buffer and the plasma-treated MMIHC device having four reaction channels (attached under the upper plate) was put on the tissue and then aligned (Figure 1C). The MMIHC microchannels were filled with the buffer and any creation of microbubbles was not allowed by the process. To avoid any leakage from microchannels, a weight was mounted on the upper plate, which the tissue was pressed by the walls of microchannels and fluids were perfectly flowed along the microchannels (Figure 1D).

Initially, deformation of reaction channels was doubted for pressure because of elastic characteristic of PDMS, which might cause different flow velocity profile for a reaction channel width. However, *z*-stacked images obtained via confocal laser scanning microscopy (LSM 510, Carl Zeiss, Germany) showed that the reaction channels retained their original rectangular shape reasonably well, and were completely separated between each channel at 8 kPa (Figure S1A).

Tissue sample intactness was verified by white light scanning interferometry (Pemtron Co., Korea). Although the tissue area attached to the MMIHC device was damaged, tissue within the reaction channels was intact; this intact region was the staining area for scoring (Figure S1B). After characterization of the MMIHC device, fluidic control for various solutions and biomarkers was conducted. Even with a reversible seal, fluid flow was perfectly controlled; at a flow rate of 300 µl h^−1^, solutions were flushed completely from the reaction channel after only 5 s. No leakage or bubble formation was observed even when the device was located on a cell block. De-waxing in xylene, rehydration, and heat-induced epitope retrieval (HIER) were conducted off-chip, whereas most of antibody−antigen interaction steps were performed on-chip. To enclose the stained tissue sample, dehydration and mounting processes were conducted after separating the MMIHC device from the slide.

### Multiplexed IHC on a Cell Block

**Figure 2:**
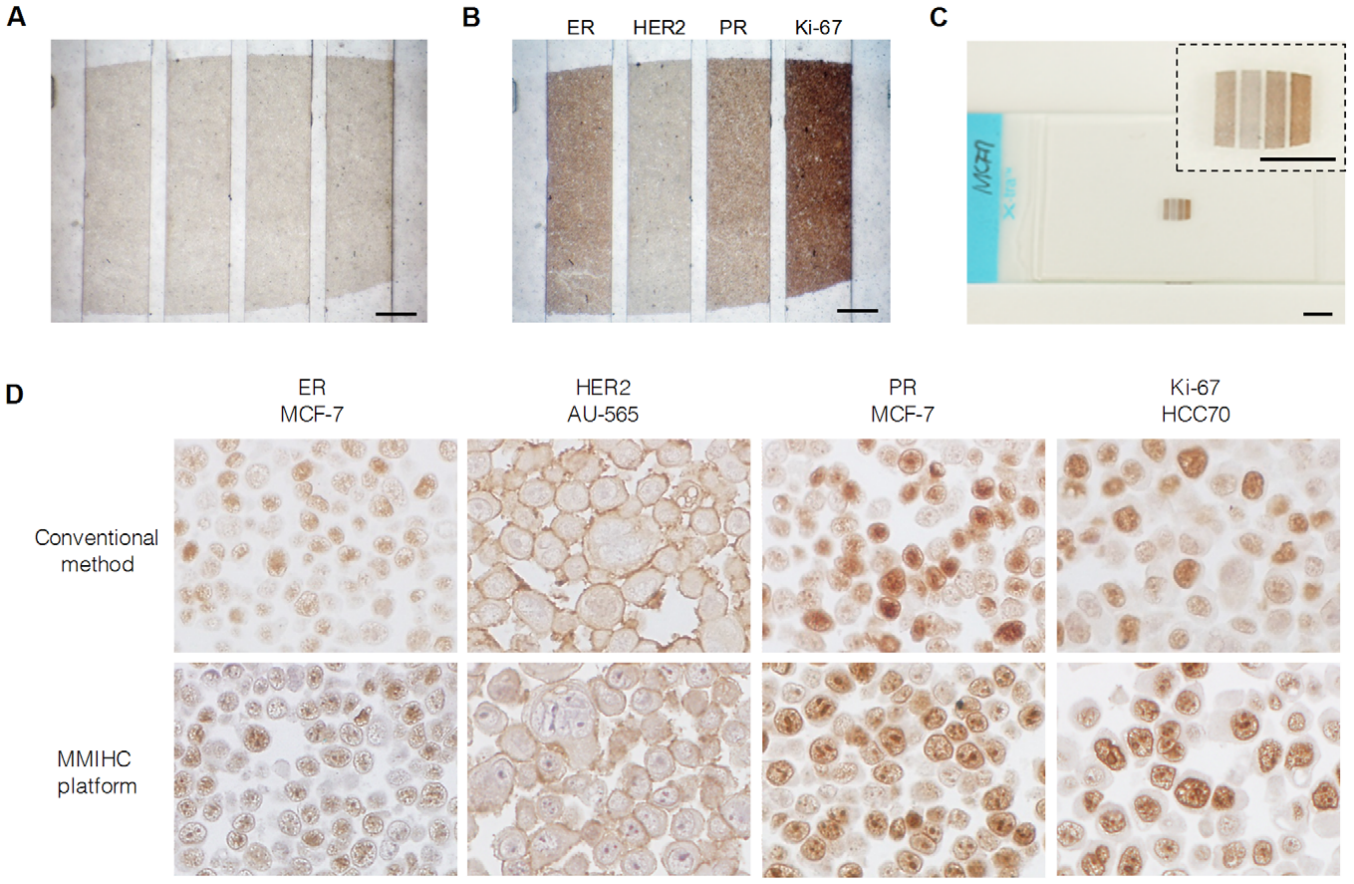
MMIHC using cell blocks. (**A**) Image of an MCF-7 cell block aligned with the MMIHC device. Scale bar, 500 µm. (**B**) Image processing DAB reaction. The cell block was stained at discrete sites with ER, HER2, PR, and Ki-67 antibodies. Scale bar, 500 µm. (**C**) Completed cell block slide after detachment from the platform. Samples were not damaged by the detachment process. Inset shows a magnified view. Scale bars, 3 mm. (**D**) Comparison of immunohistochemical staining for ER, HER2, PR, and Ki-67 using conventional IHC *versus* the MMIHC platform (1000×). The staining quality of biomarkers using the MMIHC platform was comparable to that of the conventional method.

After aligning with a cell block (Figure 2A) and injecting various solutions and biomarkers into the MMIHC device, IHC was conducted on the chip. Four biomarkers, including ER, HER2, PR, and Ki-67, were examined simultaneously on a MCF-7 cell block. Each biomarker showed a different expression level, and the pattern of immunohistochemical staining was equivalent to the geometry of the reaction channels (Figure 2B). Because the reaction channels were completely separated for each biomarker, we were able to use the same label solution (containing 3,3′-diaminobenzidine tetrachloride [DAB]) for visualization of immunological reactions for all biomarkers. Thus, parallel multiplexing allowed rapid immunohistochemical staining and a direct comparison of staining between biomarkers at one site. In addition, this approach eliminated potential variation that may occur as a result of multiple IHC steps. After counterstaining on the chip, the slide was separated from the device without any damage to the sample, and then stored using a conventional slide storage procedure (Figure 2C).

**Figure 3:**
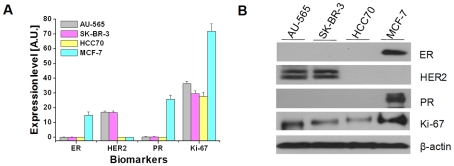
Quantification of the MMIHC method. (**A**) Quantitative comparison of biomarker expression levels in AU-565, SK-BR-3, HCC70 and MCF-7 breast cancer cell lines; mean ± SD of four replicate assays per sample. (**B**) Western blotting of AU-565, SK-BR-3, HCC70 and MCF-7 cell lines for four biomarkers.

Four breast cancer cell lines (AU-565, SK-BR-3, HCC70, and MCF-7) were examined for ER, HER2, PR, and Ki-67 expressions. All breast cancer cell lines showed staining for the indicated biomarkers at the appropriate cellular locations, providing comparable results to those obtained via conventional IHC (Figure 2D). In addition, biomarker staining was compared quantitatively for each cell line using image analysis. Microscopic images were analyzed based on the expectation-maximization (EM) algorithm and the Gaussian mixture model (GMM) to distinguish staining cells. Staining was presented as the expression level, the value of which was determined by multiplying the ratio of the stained area and the average staining intensity. ER and PR were expressed only in the MCF-7 cell line and HER2 was expressed in AU-565 and SK-BR-3 cell lines. In contrast, Ki-67 was expressed in all cell lines (order of decreasing intensity: MCF-7, AU-565, SK-BR-3, and HCC70; Figure 3A). IHC is generally regarded as a qualitative method [13], [32]; therefore, to validate the quantitative ability of the MMIHC platform, the results obtained above were compared to Western blotting results (Figure 3B). Western blotting showed that ER and PR were expressed only in the MCF-7 cell line, whereas HER2 was expressed in AU-565 and SK-BR-3 cell lines and both expression levels were similar as shown in MMIHC result. Ki-67 was expressed in all cell lines, similarly to the result that came from using the MMIHC platform; regression analysis showed that the correlation coefficient between Western blotting and MMIHC was 0.964. Overall, the expression ratio obtained via Western blotting was consistent with our results for the MMIHC platform and those of previous studies [33].

MMIHC had better repeatability than IHC for both automatic and manual staining. To compensate systemic environment and condition for MMIHC, manual IHC and automatic machine, the expression levels were normalized by their mean value of each biomarker (data not shown). The Levene's test, one of the tests for equal variance, was employed to compare the variances of normalized expression levels for above three methods (with significance level 0.05). The MMIHC had smaller variance than IHC for automatic staining (Levene's test: *p*-value = 0.044). Although the IHC for automatic staining had smaller variances than for manual method, it was not statistically significant (Levene's test: *p*-value = 0.595). This enhancement was likely the result of automated staining within the confined environment of a microfluidic system.

The MMIHC platform not only saved time and reagents, but also improved efficiency of antibody−antigen reaction. The semi-automated microfluidic platform completed IHC for the four biomarkers within 90 min, which was a 10-fold decrease in time required compared to conventional methods. In addition to time and volumetric effects, the optimal antibody concentrations were also approximately 10-fold lower. Although equivalent samples were used, staining intensity remained similar to that obtained via conventional methods even when the biomarkers were diluted 10-fold. To better understand this phenomenon, we conducted computational fluid dynamics (CFD) studies on the kinetics of receptor−ligand binding. Under conventional IHC conditions, although the original concentration was maintained on the tissue in the initial state, the concentration of analyte exposed to the tissue was significantly lower because the analyte in the vicinity of tissue was bound to tissue receptors. In contrast, the concentration of analyte showed very little change at the tissue surface when the analyte was allowed to flow through the MMIHC platform. Because the reaction rate of receptor−ligand binding is decided by the absolute concentration of the analyte, and because mass transport to an area with very low fluid velocity (*i.e.*, such as that near to the tissue sample) is determined by the concentration gradient, a high Reynolds number (*Re*) likely resulted in the continuous exposure of the initial analyte concentration and the formation of a steep concentration gradient (Figure S2).

**Figure 4:**
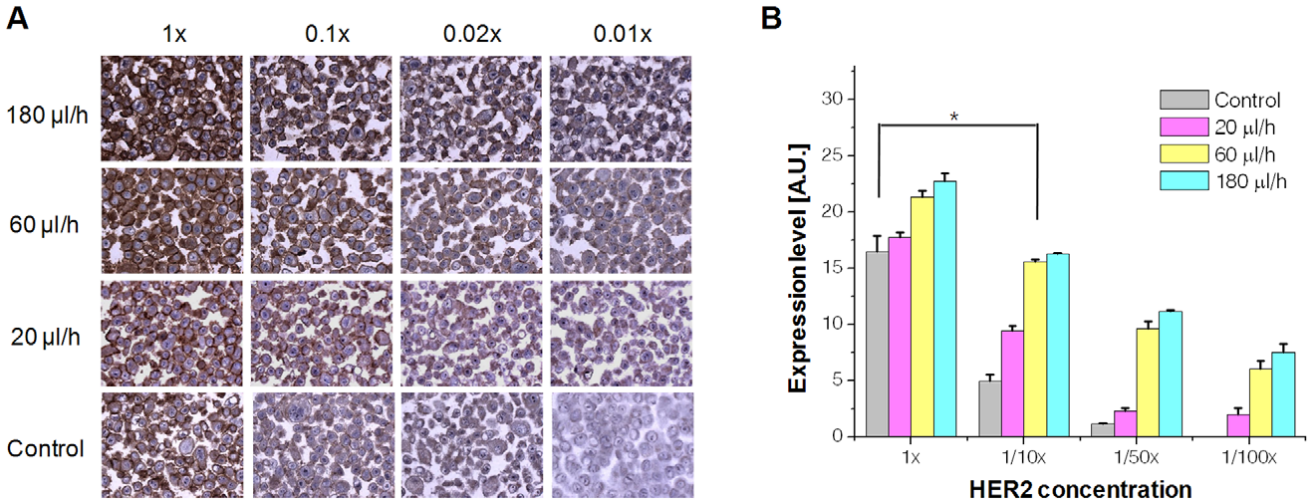
Staining efficiency as a function of incubation conditions. (**A**) Staining results for the SK-BR-3 cell line according to changes in flow rate and HER2 antibody concentration (incubation time, 30 min). Stronger expression was observed at higher flow rates. (**B**) Quantitative evaluation of HER2 expression under static and dynamic incubation conditions. As shown in the graph, the expression level of the 1× HER2 control (conventional method) was not significantly different to that of 0.1× HER2 at a flow rate of 60 µl h^−1^. The asterisk denotes statistically no significant difference (*p* = 0.395) between the indicated pair.

We also showed that staining was more intense after the same incubation period when the flow rate was high (Figure 4A). Quantification clearly showed that higher flow rates produced higher expression levels for HER2 (Figure 4B). In addition, expression levels at flow rates of 60 and 180 µl h^−1^ when using a concentration of 0.1× HER2 were similar to those obtained using a conventional IHC method at a concentration of 1× HER2; this low concentration corresponded to the optimal antibody concentration determined by a pathologist. Based on these results and those of several other trials, we determined the optimal incubation conditions for primary antibodies (flow rate, 100 µl h^−1^; incubation period, 10 min), which translate into a >6-fold decrease in required time and 200-fold decrease in antibody consumption.

### MMIHC for Human Breast Cancer Tissues

After verifying the utility of MMIHC on a chip and examining repeatability and the possibility of quantification using cell blocks, we applied the platform to patient tumor tissues which are heterogeneous in terms of morphology, genetic alterations and histopathological features. Over one hundred cases of human breast tumor tissues (115 cases) were randomly selected and the distributions of the investigated biomarkers were not biased. All experiments, including the MMIHC operation and clinical analysis, were blindly conducted. A pathologist judged the cases did not know not only the whole-section results of the cases but also any information of cases tested by the MMIHC platform.

**Figure 5:**
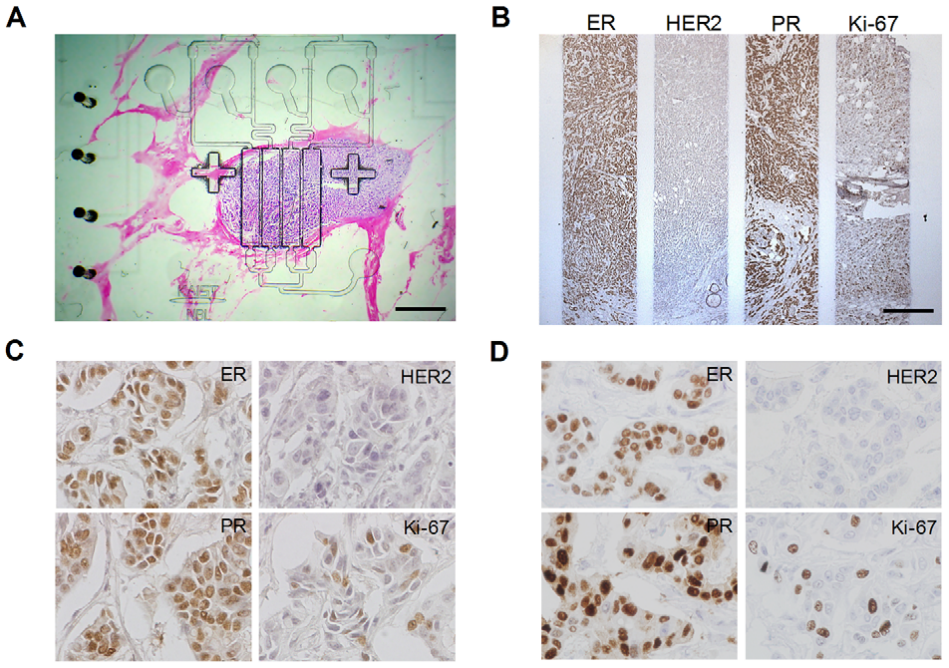
Application of the MMIHC platform to human breast cancer tissues and comparison with conventional IHC. (**A**) The alignment of MMIHC device with an H&E-stained tissue sample. The device was aligned at a site showing numerous stained nuclei, indicating an area containing proliferating cancerous cells. Scale bar, 3 mm. (**B**) MMIHC image for a human breast cancer tissue sample. Compared to conventional IHC, the image shows the expression of the four biomarkers (ER, HER2, PR, and Ki-67) simultaneously on a tissue slide. Scale bar, 500 µm. (**C**) Magnified images of panel b (×400). (**D**) Images of ER, HER2, PR, and Ki-67 staining via conventional IHC. The expression of biomarkers and the assigned Allred scores were equivalent.

Unlike cell blocks, tissue samples are not homogeneous; therefore, aligning the MMIHC device over the least heterogeneous area is critical for diagnostic outcome. The device was aligned at the area of highest cancer cell density, which was determined with the use of hematoxylin and eosin (H&E) staining slide (Figure 5A). After that, we examined the biomarker expression. Four biomarkers were examined simultaneously on the same tissue sample. In contrast to the cell blocks, which showed homogeneous staining patterns, it was difficult to distinguish exact areas of biomarker staining because of tissue heterogeneity and some weak or negative staining results. This was resolved by injecting Mayer's hematoxylin into the microchannels, which clearly demarcated the reaction channels (Figure 5B). Staining for the biomarkers was localized properly and score values for biomarkers were also equivalent to that obtained via conventional IHC (Figure 5C and 5D). Especially for PR, more non-specific staining was shown in conventional automatic IHC machine (Figure S3).

Although the biomarkers were examined in the areas in which cancer was most severe, a comparative study was essential to clarify whether the results from such a localized examination using the MMIHC platform could be considered representative of the whole tissue section. A blind experiment was performed and the 105 patient tissue slides investigated via MMIHC were scored by a pathologist (Table S1). The results revealed that Kendall's coefficient of concordance (KCC, *n* = 105) was 0.96 for ER, 0.90 for HER2, 0.95 for PR, and 0.98 for Ki-67; the agreement rates (*κ* coefficient, *n* = 105) were 0.92, 0.65, 0.79 and 0.87 for ER, HER2, PR, and Ki-67, respectively (Table 1). HER2 showed the lowest KCC, although it should be noted that many cases for mismatches were slight (scores of 0 *versus* 1+). PR also showed a lower match rate than ER, which is consistent with previous studies showing that PR had lower sensitivity, specificity, and overall *κ* values than ER [34].

**Table 1:** Statistical concordance data for whole-section analysis *versus* the MMIHC platform (*n* = 105), including Kendall's coefficient of concordance (KCC), χ^2^ test, *κ* statistics, concordance and its 95% confidence interval (CI).

	Kendall's coefficient of concordance (KCC)	χ^2^	*p*-value	*κ*-value	*p*-value	% Concordance	95% confidence interval (CI)
ER	0.96	200	<0.0001	0.92	<0.0001	98.1	93.3−99.4
HER2	0.90	187	<0.0001	0.65	<0.0001	85.0	76.4−91.0
PR	0.95	198	<0.0001	0.79	<0.0001	90.5	83.2−95.3
Ki-67	0.98	204	<0.0001	0.87	<0.0001	91.4	84.4−96.0

The KCCs were 0.96 for ER, 0.90 for HER2, 0.95 for PR, and 0.98 for Ki-67, and the agreement rates (*κ* coefficient) were 0.92, 0.65, 0.79, and 0.87 for ER, HER2, PR, and Ki-67, respectively. It is noted that KCC values >0.90 are regarded as almost perfect degree of agreement [45] and *κ* values >0.61 are considered as substantial agreement [34].

**Figure 6:**
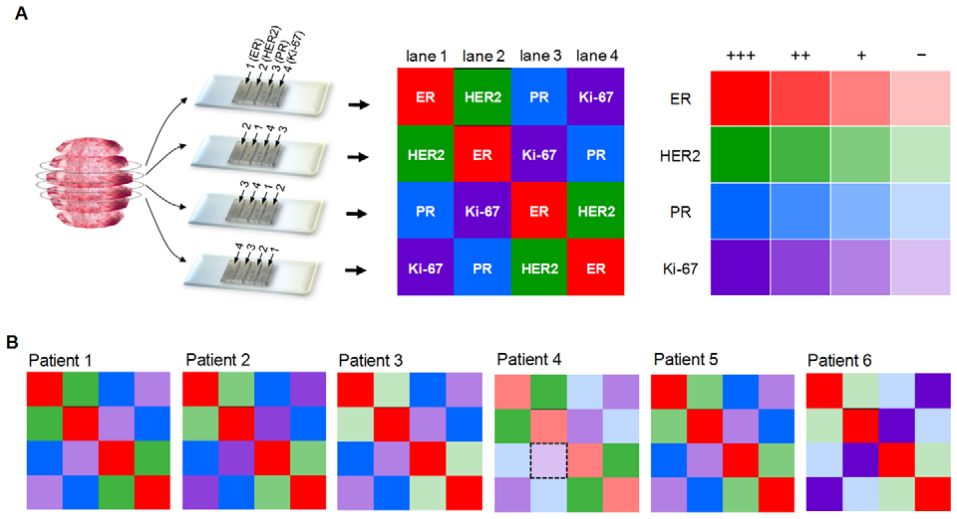
Reproducibility study within tissues originated from the same patient. (**A**) Four slides were made from the human breast tumor of the same patient and cyclic permutation tests were conducted; ER, HER2, PR and Ki-67 for 1st slide, HER2, ER, Ki-67 and PR for 2nd slide, PR, Ki-67, ER and HER2 for 3rd slide, and Ki-67, PR, HER2 and ER for 4th slide. (**B**) Results of cyclic permutation tests for six cases. When the intensity of each color is equivalent in the boxes, the case was perfectly matched. The black dotted square for Patient 4 denotes different intensity compared to others.

After confirmation of concordance between whole tissue section analysis and the MMIHC platform, we conducted the reproducibility study whether the platform also showed the same results within tissues originated from the same patient. Six cases were tested for reproducibility where four slides were made from the breast tumor of the same patient. To verify the concern of tissue heterogeneity with reproducibility, for each case, a simple Latin square of order 4 was used by cyclic permutation tests in the first slides for subsequent slides to eliminate the sequential effect of tests; ER, HER2, PR and Ki-67 for 1st slide, HER2, ER, Ki-67 and PR for 2nd slide, PR, Ki-67, ER and HER2 for 3rd slide, and Ki-67, PR, HER2 and ER for 4th slide (Figure 6A). The biomarkers and their score levels were expressed with colors and their intensity (Figure 6B). The KCCs within appraiser showed that all biomarkers had over 0.95 values by repetition of measurement as all *p*-values are sufficiently small (Table S2). This result indicates that the platform is also reproducible in tissue sample and the sequence of the biomarkers does not affect score results significantly.

Not all tissue samples were suitable for the MMIHC platform. Although most tissues were firmly attached to the slides, tissue detachment occasionally occurred after HIER. In total, about 9% of the slides showed tissue detachment (10 cases among 115 samples). This problem is likely resolvable through optimization of the sample preparation process, including fixation, sectioning, and drying.

### MMIHC on a Needle Biopsy Sample of Human Breast Cancers for Precise Diagnosis in Early Stage

**Figure 7:**
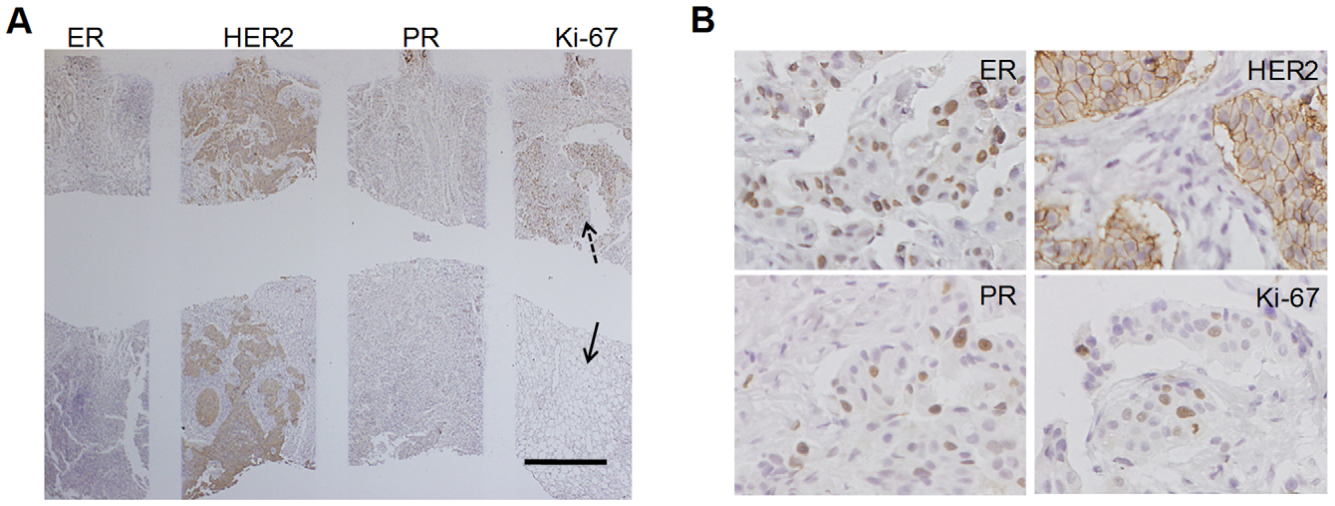
MMIHC on a needle biopsy sample of human breast cancers as a model of scarce tissue samples. (**A**) MMIHC image of a tissue from needle biopsy of human breast cancers. The solid arrow indicates fatty tissue that showed less intense staining compared to non-fatty tissue (dotted arrow). Scale bar, 500 µm. (**B**) Images of ER, HER2, PR, and Ki-67 expression in needle biopsy tissue (400×). Each biomarker was properly stained and the result was consistent with those obtained via conventional IHC.

Preoperative chemotherapy has been used for large primary and inflammatory breast cancers, and the examination of specific biomarkers in needle biopsy samples greatly facilitates the early individualization of neoadjuvant therapy [35]. Therefore, we applied the platform on a tissue from needle biopsy of human breast cancers, which reduced consumption of the rare tissues and enabled investigation for more various biomarkers even in small-sized samples. Despite the narrow area of core biopsy samples, the MMIHC device was aligned easily and we were able to examine the expression of four biomarkers on a single slide (Figure 7A). We noted that fatty tissue (solid arrow in Figure 7A) should be avoided when selecting an inspection window; biomarker expression in such regions was inconsistent compared to other non-fatty areas (dotted arrow in Figure 7A). Similar to cell blocks and tissue samples, the four biomarkers were also expressed in needle biopsy tissues (Figure 7B). A concordance study showed that the agreement rate (Cohen's *κ* coefficient, *n* = 8) was 1 for ER (*p* = 0.0001), 0.71 for HER2 (*p* = 0.0175), and 0.73 for PR (*p* = 0.0044). In the case of tissues from needle biopsy, the *κ* statistics showed substantial agreement in concordance and ER showed 100% concordance and HER2 and PR showed 87.5% concordance (data not shown).

## Discussion

The MMIHC platform, which was realized IHC on a chip and was applied to human clinical tissues specimens for the first time, minimized the use of externally connected equipment and formed a simple interface with the tissue sample. The unique platform significantly reduced the probability of assay failure (under 1%), which is of critical importance in practical use when dealing with clinically rare samples. Because the use of microfluidic channels creates a confined microenvironment and staining was semi-automated, MMIHC showed better repeatability of immunohistochemical staining compared to conventional manual IHC method and automatic IHC machine. This benefit seems to give improvement for inconsistent problem of IHC. Furthermore, the MMIHC yielded a 200-fold reduction of antibody consumption, fast immunological reaction, and the ability to examine various biomarkers for cancer chemotherapy in rare tissue samples. The characteristics of MMIHC platform are expected to reduce costs required for examination of various biomarkers when it is fully developed with a full automated and high-throughput manner.

Quantitative scoring is one of the main topics to overcome scoring subjectivity immunohistochemical analysis. By comparing our results with those obtained via Western blotting, we showed that the MMIHC platform is suitable for the semi-quantitative analysis of cell blocks. In addition, since MMIHC enabled the direct comparison of biomarker staining at a single site and eliminated the unexpected variation that may arise from multiple IHC steps, more accurate relative quantification was expected. Because the inspection window for each biomarker is relatively small, it would be also beneficial to perform quantification with image analysis as same rationale with TMAs [36]. To adopt automated quantitative image analysis into tissue results, however, staining between ductal carcinoma in situ (DCIS) and invasive carcinomas should be distinguished. Therefore, additional advances should be made in extensive image analyses and improved algorithms to decide clear scores.

Although this platform has many advantages over the conventional and automated machine-based IHC methods, it was doubted whether the MMIHC results are consistent with those obtained via conventional whole-tissue section analysis. Actually, this was also a critical concern regarding the use of TMA, which uses small tissue cores that may not be representative of the whole tissue section. However, after publication of the initial TMA results, many subsequent studies have shown excellent correlation for various tumor types [37]−[39]. Because the reaction channel of the MMIHC device was 14-fold wider than the 600-µm TMA core, it was expected to correlate well with the results of conventional methods, although TMAs are advantageous in their own right for permitting the selection of several tissue cores and thus enhancing representativeness. As we expected, in statistical aspect, results showed all of biomarkers showed over 0.90 KCC values, reflecting the whole section IHC scores with almost a perfect degree of agreement. This tendency was also shown in tissue samples from needle biopsies.

In clinical aspects, the MMIHC method is likely to be no detriment to patient care in clinical settings. Although there are many reasons for discrepancy of scores between whole-section analysis and MMIHC such as inborn errors with IHC itself (intraobserver and interlaboratory variations), specimen selection, processing and representativeness of MMIHC result, total cases showing discordance in this study were 34 cases. Among our 34 discordant results, there were only two cases (case #27 and 93) of disagreement between control and MMIHC for ER which did not result in change of treatment plans (2+ = >3+). For PR results, three cases (case #7, 86, and 103) would result in a difference when making treatment decisions. However, only one case (case #103) could actually be treated differently since the ER status is always considered simultaneously. The other seven cases (case #18, 20, 30, 39, 59, 60, and 68) did not result in any change of treatment plans (1+ = >2+ or 3+ = >2+). Most of the discordance occurred in HER2 assessment. Specifically, two cases (case #28 and 69) among 16 discordant cases had potential risk for receiving trastuzumab. In case #69, since HER2 2+ by automated conventional method should be subsequently tested by fluorescence in situ hybridization (FISH) and treatment is dependent on the FISH result. Three cases (case #26, 100, and 101) would require additional FISH test. In summary, the cases that cause clinically different treatment are 0 case for ER, 1 case for PR and 1 case for HER2, showing only 1.9% variation in clinical treatment. Therefore, on the basis of our results, the MMIHC platform showed sufficient possibility of adoption as a method for the presentation of clinical specimens.

Another concern was the different scores depending on the sequence of biomarkers owing to tissue heterogeneity. Cyclic permutation test, however, revealed that scores of the biomarkers were reproducibly repeated and the sequence of the biomarkers did not affect score results significantly. This result is also likely to imply that the aligning position is not such extremely critical; meaning that the slight different position at the area of highest cancer cell density was affordable in reproducibility. Although this study was conducted in a microfluidic device with 800-µm-wide reaction channels to satisfy so much as tissues having small cancer core, increasing the width and number of the channels in different directions is expected to enhance the representativeness of the results, in the same way that the concordance of results obtained via TMA was improved by increasing the number and size of the cores [39]−[41].

In improving the standard of patient care, many issues must still be resolved before the current format of the MMIHC platform can compete with existing methods. Although the MMIHC approach contains a sampling process within a whole section of tissue, it is obvious that an assay that could accurately quantify several cancer-related proteins simultaneously on single tumor section or small tumor specimens does offer clear advantages and technical advances over standard immunohistochemical method [21]. Therefore, a novel method showing substantial agreement for KCC statistics is expected to be useful as a decision supporting tool for pathologist and clinician.

In IHC, multiplex staining is growing need within limited quantity of clinical samples. Many studies for multiplex IHC have been based on the multicolor approach [18]−[21]. Sequential indirect multiplexing method, which conducts blocking, antibody reaction and then tagging with fluorescence in repeat, has an advantage to look at a wide range of co-expressions for biomarkers. However, it is a labor intensive and time consuming process, and might cause a cross-over nonspecific binding of secondary probes as long as the serial processing was increased [23]. In addition, it is not suitable for high-throughput purpose. These problems can be solved by direct staining method, which each primary antibody is conjugated to a probe showing a different color and a mixture of the probe conjugates was exposed to a tissue sample in a single step. Although the approach reduces time and labors, undesirable problems are confronted: 1) some primary antibodies are not sometimes survived in the probe conjugation process and their binding properties are often changed. 2) Probe conjugation to primary antibodies is hard when the original antibody buffer contains serum or other proteins. 3) Reagent costs can be considerably high whose issue prohibits clinical application [22]. On the contrary, the parallel multiplexing method introduced in this study significantly compensates direct and indirect sequential multiplexing methods, so that it has several advantages: 1) reduction of time consumption and labor intensiveness, 2) original elimination of cross-over nonspecific tagging between biomarkers, 3) free usability of conventional primary antibodies, and 4) cost-effectiveness. In addition, the method has a potential to enhance throughput when the direct or indirect sequential method was combined. Moreover, to address the multiplexing and *in situ* quantification of this new technology, tens of IHC assays are possible on a specimen once the number of reaction channels is increased. It implies that the MMIHC platform may be useful to confirm and complement the results of similarly scaled genomic assays, such as the Oncotype DX test (which also examines 21 factors), which will help to understand the heterogeneous and complicated phenomena associated with cancers.

The development of TMAs in 1998 [42] significantly accelerated the process of validating biomarker expression in clinical samples [43]. Although TMAs have many advantages over conventional manual IHC methods, direct application to clinical diagnosis has been restricted and TMA has remained a research tool because of practical limitations, such as the requirement of fast diagnostic results for urgent patients, sample damage, and tedious procedures for tissue collection. However, our platform is almost compatible to conventional IHC using a single thin-sectioned tissue slide. Therefore, it is expected to support various potential prognostic markers in clinical stage. As another aspect, because MMIHC is essentially complementary to TMA (*i.e.*, multiple channels connected on top of a tissue slide rather than multiple cores taken from different tissue samples on a single slide), it may compensate for limitations associated with the latter method. For example, by TMA use, current study has revealed that 27 IHC markers proved to be significant prognostic indicators for 924 patients to predict disease outcome [44]. When the significant indicators are investigated in research domain by TMAs, they are practically able to be applied to patients by the platform. In addition, it is expected to be a more effective and high-throughput tool if the platform is combined with a TMA tissue slide that has 4-mm core punctures. We anticipate a simple, fast, and quantitative MMIHC platform will be broadly applicable to clinical diagnosis, identification of novel markers for classifying solid tumors, the selection of optimal biomarkers, development and screening of IHC antibodies, biological pathway studies and establishment of optimal IHC conditions (*e.g.*, antibody concentration and incubation time) in various cancers.

## Materials and Methods

### Ethics Statement

Human tissue samples from each tumor lesion were obtained from the National Cancer Center Hospital (Goyang, Korea) and the Korea University Anam Hospital (Seoul, Korea), with the corresponding written consents given by the patients or their relatives. This study was approved by the Institutional Review Board (IRB) at the National Cancer Center Hospital and Korea University Anam Hospital.

### Fabrication of a MMIHC Device

The fluidic channel mold for a MMIHC device was fabricated via two-step multilayer soft lithography. To construct rectangular reaction channels, SU-8 2025 (Microchem Corp., MA) was spin-coated to form a 50-µm thick layer on a bare silicon wafer, patterned by UV exposure. After developing the wafer, a masking layer was patterned on the reaction channel area. To make a round-shaped remnant fluidic channel, AZ 9260 was spin-coated to form a 25-µm thick layer. After lifting off the masking layer, it was exposed to UV light, and developed using AZ photoresist developer. The fabricated mold was reflowed by heating, and the fluidic channels were transformed into a round shape, except for the reaction channels. The control channel mold was fabricated by conventional SU-8 photolithography. After spin-coating the fluidic channel mold with PDMS and curing, the fluidic channel was aligned and bonded with the control channel using an O_2_ plasma asher (270 W for 30 s).

### Preparation of Cell Blocks and Tissues

Four commercially available breast carcinoma cell lines, MCF-7, SK-BR-3, AU-565, and HCC70, were obtained from the American Type Culture Collection (ATCC; Manassas, VA). HCC70, MCF-7, and AU-565 were maintained in RPMI-1640 and SK-BR-3 cells were maintained in Dulbecco's modified Eagle's medium (DMEM) supplemented with 10% fetal bovine serum (FBS), 100 IU ml^−1^ penicillin, and 100 mg ml^−1^ streptomycin. All cell lines were cultivated at 37°C and incubated in 5% CO_2_. Adherent cells were harvested by trypsinization before reaching confluence. For IHC analysis, the harvested cells were centrifuged, fixed in formalin, suspended in agar, and embedded in paraffin to produce a cell block. Paraffin-embedded cell blocks were sectioned at 4 µm thickness using a microtome (Leica, Germany). The sections were baked onto positively charged slides and allowed to dry for 1 h at room temperature, followed by 1 h in an incubator at 60°C.

Tissue samples from each tumor lesion were fixed for 24 h in 4% neutral-buffered formalin, Bouin's fixative, acetic formalin alcohol (AFA), or 4% or 10% unbuffered formalin; 4 h in PreFer (Anatech, Battle Creek, MI) or Pen-Fix (Richard Allen Scientific; Kalamazoo, MI); or 48 h in 4% neutral-buffered formalin. After paraffin embedding, tumor specimens were cut into 4-µm-thick sections and allowed to dry for 1 h at room temperature, followed by 1 h in an incubator at 60°C.

### Immunohistochemical Staining

Four biological markers were investigated. ER (SP1) antibody (Ventana, Tucson, AZ) and PR (1E2) antibody (Ventana) were used in conventional methods at 1× concentration and in MMIHC at a dilution of 1∶10. HER2 oncoprotein antibody (Dako, Denmark) was used in conventional methods at a dilution of 1∶500 and in MMIHC at a dilution of 1∶5000. Ki-67 (clone MIB-1) antibody (Dako) was used in conventional methods at a dilution of 1∶50 and in MMIHC at a dilution of 1∶500. Cell blocks and tissues were de-waxed in xylene and rehydrated through a graded series of ethanol solutions. A microwave antigen-retrieval technique was used and the samples were treated in target retrieval solution, pH 9 (Dako), for 20 min at 750 W. The Cap-plus kit (Zymed, San Francisco, CA) was used for immunostaining and Mayer's hematoxylin (Sigma, St Louis, MO and Labvision, Fremont, CA) was used for counterstaining.

### Western Blotting

Protein was extracted from cells by the addition of lysis buffer followed by centrifugation at 16,000 g for 10 min at 4°C. The supernatant fractions were separated by polyacrylamide gradient gel (8–16%) containing sodium dodecyl sulfate. Following electrophoresis, the proteins were transferred to polyvinylidene fluoride membranes (Millipore, Bedford). The membranes were blocked in TBS-T (0.1% Tween 20) containing 5% non-fat milk (Bio-Rad, Richmond) for 1 h at room temperature. After blocking, the membranes were incubated for 2 h at room temperature with primary antibodies against ER, HER2, PR, and Ki-67 (clone MIB-1) antibodies. Then, the membranes were washed in TBS-T (0.1% Tween 20), for 15 min at a time, and incubated with diluted HRP-conjugated secondary antibody (Southern Biotech, Birmingham, AL) for 1 h at room temperature. This was followed by washing with TBS-T (3×15 min), incubation with WEST-ZOL® plus chemiluminescence reagent (iNtRON Biotechnology, Korea) for 1 min, and exposure to film (Kodak Blue XB-1, Rochester, NY). The immunoblot of β-actin (R&D Systems, Korea) was used as a loading control.

### Image Acquisition and Analysis for Quantification of MMIHC

Tissue images were taken in optimal condition considering shading and glare. After completing MMIHC, the sample was placed on an inverted microscope (Carl Zeiss, Germany) and images were acquired by a microscope CCD camera (Olympus DP71, Japan) under 13,000 Lux light intensity. The microscope CCD camera has 12.5 megapixels, 12-bit digital color that displays the native CCD's full-resolution live image at 15 frames per second. The microscopic images were divided into three parts (Figure S4A): the staining part (SP) of the cell, the non-staining part (NSP) of the cell, and the background (Figure S4B). Then the expression level was defined by multiplying the staining ratio and intensity, where the staining ratio is the area ratio of the SP to the cell part (SP and NSP) and the staining intensity is the average intensity in the SP. At least five images were randomly taken along the individual reaction channel via 400× magnification and the average expression level for the images was presented as a representative value of immunohistochemical staining for a biomarker.

Bayesian classification was employed to segment a microscopic image into the three parts based on their colors. The color distribution of each part in RGB color space was represented by the Gaussian mixture model (GMM), and estimated by the expectation-maximization (EM) method. The probability density function (PDF) of GMM with *C* components is defined as a convex combination of Gaussian PDFs
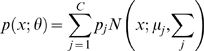
(1)where *N*(*x*; *μ*, *∑*) is the d-dimensional Gaussian PDF with mean *μ* and the covariance ∑, and *p_j_* is the portion of the j-th component such that 0<*p_j_*<1 for all components, and 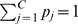. The parameter list
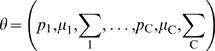
(2)defines a particular PDF of the GMM. The parameters for the SP, NSP, and background were obtained via the EM method using data collected from IHC sample images. The numbers of clusters in the GMM for the SP, NSP, and background were set at 5, 10, and 2, respectively. Thirty independent IHC images were used to train the PDF. The optimized Gaussian PDF was automatically applied to the experimental images.

### Data and Statistical Analyses

We compared the quantification of ER, HER2, PR, and Ki-67 expressions between MMIHC and the respective quantified Western blot bands by using Pearson's correlation. For ER and PR, the Allred scores (0−8) assigned by a pathologist were translated into negative (−) for 0 score, weak (+) for 2 and 3 scores, intermediate (++) for 4, 5, and 6 scores, and strong (+++) for 7 and 8 scores. For HER2, we followed the HER-2/neu FDA-approved scoring system and translated into (−) for 0, (+) for 1+, (++) for 2+, (+++) for 3+. For Ki-67, stained cell number was counted and translated into (−) for ≤5%; (+) for 5%<*x*≤20%; (++) for 20%<*x*≤40%; (+++) for >40%. Attribute agreement analysis for tissue data was performed using Minitab version 15. The relative agreement between conventional IHC and MMIHC data for ER, HER2, PR, and Ki-67 was assessed using KCC. Fleiss' and Cohen's *κ* statistics were calculated to evaluate the agreement between the two systems, for which the contingency tables between conventional IHC and MMIHC for ER, HER2, PR, and Ki-67 are presented. Hypothesis testing was conducted using the two sample *t* test to analyze HER2 and Ki-67 expression in the four breast cancer cell lines.

## Supporting Information

Table S1 Comparison of scores obtained via whole-section analysis (control) versus the MMIHC platform in human breast cancer tissues (*n* = 105). For ER and PR: negative (−); weak (+); intermediate (++); strong (+++). For HER2: score 0 (−); score 1 (+); score 2 (++); score 3 (+++). For Ki-67: ≤5% (−); 5%<*x*≤20% (+); 20%<*x*≤40% (++); >40% (+++). The unit of tumor size is centimeter. DCIS, ductal carcinoma in situ.
(0.92 MB DOC)

Table S2 Statistical concordance table of cyclic permutation test, including KCC, χ^2^ test and *p*-value. Six cases were tested for reproducibility where four slides were made from the breast tumor of the same patient (total number of tissues: 24). The KCCs were 1.00 for ER, 1.00 for HER2, 1.00 for PR, and 0.96 for Ki-67, respectively. The cyclic permutation tests indicate that the MMIHC platform is reproducible in tissue sample and the sequence of the biomarkers does not affect score results significantly.
(0.76 MB DOC)

Figure S1 Characterization of the MMIHC platform. (A) Plane and *z*-stacked confocal laser microscopy images of the reaction channel area under 8 kPa. Reaction channels retained their original rectangular shape and each was separated completely. (B) Surface image of a cell block visualized using white light scanning interferometry. Cells in the reaction channels were intact under pressure, except for those in areas in direct contact with the MMIHC device.
(0.25 MB TIF)

Figure S2 Computational fluid dynamics (CFD) study examining the kinetics of receptor-ligand binding. (A) Concentration profile of the analyte using the conventional method (transient state after 80 s). The red dotted line indicates a tissue sample with antigens. The analyte concentration in the vicinity of the tissue decreased with time because the tissue functioned as a sink. (B) Concentration profile of the analyte using the MMIHC platform (*Re* = 4.3; transient state after 80 s). Fresh analyte flowed into and was maintained in the vicinity of the tissue; therefore, the concentration of the analyte showed little decrease at the tissue surface as time progressed. (C) Concentration distribution of the analyte according to incubation conditions. The concentration profiles between a non-flowing microchannel and the conventional method were similar, and the concentration of the analyte exposed to the tissue was higher when the flow velocity of the analyte increased. (D) Concentration gradient versus analyte incubation conditions. When the flow velocity was high, diffusion of the analyte was dominant.
(0.21 MB TIF)

Figure S3 Images of immunohistochemical staining for PR. (A) An image of PR staining from automatic machine (×400). (B) An image of PR staining from the MMIHC platform (×400). Blue solid arrows indicate non-specific staining. Normally, more non-specific staining was shown in conventional automatic IHC machine.
(0.68 MB TIF)

Figure S4 Image analysis of biomarker expression level. (A) A microscopic image acquired via MMIHC. (B) The image was divided into three parts: the staining part (SP), the non-staining part (NSP), and the background. Only the cell area (SP and NSP) was considered to minimize the variation of expression level according to cell density. (C) Image after analysis. Only the brown-colored areas remained.
(0.36 MB TIF)

## References

[1] Stearns V, Schneider B, Henry NL, Hayes DF, Flockhart DA (2006). Breast cancer treatment and ovarian failure: risk factors and emerging genetic determinants.. Nat Rev Cancer.

[2] Laxman B, Morris DS, Yu J, Siddiqui J, Cao J (2008). A first-generation multiplex biomarker analysis of urine for the early detection of prostate cancer.. Cancer Res.

[3] Ludwig JA, Weinstein JN (2005). Biomarkers in cancer staging, prognosis and treatment selection.. Nat Rev Cancer.

[4] van't Veer LJ, Bernards R (2008). Enabling personalized cancer medicine through analysis of gene-expression patterns.. Nature.

[5] Dietel M, Sers C (2006). Personalized medicine and development of targeted therapies: The upcoming challenge for diagnostic molecular pathology. A review.. Virchows Arch.

[6] Wang HN, Vo-Dinh T (2009). Multiplex detection of breast cancer biomarkers using plasmonic molecular sentinel nanoprobes.. Nanotechnology.

[7] van't Veer LJ, Dai H, van de Vijver MJ, He YD, Hart AA (2002). Gene expression profiling predicts clinical outcome of breast cancer.. Nature.

[8] Paik S, Shak S, Tang G, Kim C, Baker J (2004). A multigene assay to predict recurrence of tamoxifen-treated, node-negative breast cancer.. N Engl J Med.

[9] Carey LA, Perou CM, Livasy CA, Dressler LG, Cowan D (2006). Race, breast cancer subtypes, and survival in the Carolina Breast Cancer Study.. JAMA.

[10] Rouzier R, Perou CM, Symmans WF, Ibrahim N, Cristofanilli M (2005). Breast cancer molecular subtypes respond differently to preoperative chemotherapy.. Clin Cancer Res.

[11] Sorlie T, Perou CM, Tibshirani R, Aas T, Geisler S (2001). Gene expression patterns of breast carcinomas distinguish tumor subclasses with clinical implications.. Proc Natl Acad Sci U S A.

[12] Wulfkuhle JD, Liotta LA, Petricoin EF (2003). Proteomic applications for the early detection of cancer.. Nat Rev Cancer.

[13] Walker RA (2006). Quantification of immunohistochemistry–issues concerning methods, utility and semiquantitative assessment I.. Histopathology.

[14] Lakhani SR, Ashworth A (2001). Microarray and histopathological analysis of tumours: the future and the past?. Nat Rev Cancer.

[15] Perou CM, Sorlie T, Eisen MB, van de Rijn M, Jeffrey SS (2000). Molecular portraits of human breast tumours.. Nature.

[16] Kao J, Salari K, Bocanegra M, Choi YL, Girard L (2009). Molecular profiling of breast cancer cell lines defines relevant tumor models and provides a resource for cancer gene discovery.. PLoS One.

[17] Paik S, Shak S, Tang G, Kim C, Baker J (2004). A multigene assay to predict recurrence of tamoxifen-treated, node-negative breast cancer.. N Engl J Med.

[18] Wu X, Liu H, Liu J, Haley KN, Treadway JA (2003). Immunofluorescent labeling of cancer marker Her2 and other cellular targets with semiconductor quantum dots.. Nat Biotechnol.

[19] Lidke DS, Nagy P, Heintzmann R, Arndt-Jovin DJ, Post JN (2004). Quantum dot ligands provide new insights into erbB/HER receptor-mediated signal transduction.. Nat Biotechnol.

[20] Fountaine TJ, Wincovitch SM, Geho DH, Garfield SH, Pittaluga S (2006). Multispectral imaging of clinically relevant cellular targets in tonsil and lymphoid tissue using semiconductor quantum dots.. Mod Pathol.

[21] Yezhelyev MV, Gao X, Xing Y, Al-Hajj A, Nie S (2006). Emerging use of nanoparticles in diagnosis and treatment of breast cancer.. Lancet Oncol.

[22] Xing Y, Chaudry Q, Shen C, Kong KY, Zhau HE (2007). Bioconjugated quantum dots for multiplexed and quantitative immunohistochemistry.. Nat Protoc.

[23] Sweeney E, Ward TH, Gray N, Womack C, Jayson G (2008). Quantitative multiplexed quantum dot immunohistochemistry.. Biochem Biophys Res Commun.

[24] Kim MS, Yeon JH, Park JK (2007). A microfluidic platform for 3-dimensional cell culture and cell-based assays.. Biomed Microdevices.

[25] Yeon JH, Park JK (2007). Microfluidic cell culture systems for cellular analysis.. Biochip J.

[26] El-Ali J, Sorger PK, Jensen KF (2006). Cells on chips.. Nature.

[27] Nagrath S, Sequist LV, Maheswaran S, Bell DW, Irimia D (2007). Isolation of rare circulating tumour cells in cancer patients by microchip technology.. Nature.

[28] Kim MS, Lee W, Kim YC, Park JK (2008). Microvalve-assisted patterning platform for measuring cellular dynamics based on 3D cell culture.. Biotechnol Bioeng.

[29] Yeon JH, Park JK (2009). Drug Permeability Assay Using Microhole-Trapped Cells in a Microfluidic Device.. Anal Chem.

[30] Liu GL, Kim J, Lu Y, Lee LP (2006). Optofluidic control using photothermal nanoparticles.. Nat Mater.

[31] Hahn YK, Jin Z, Kang JH, Oh E, Han MK (2007). Magnetophoretic immunoassay of allergen-specific IgE in an enhanced magnetic field gradient.. Anal Chem.

[32] Taylor CR, Levenson RM (2006). Quantification of immunohistochemistry–issues concerning methods, utility and semiquantitative assessment II.. Histopathology.

[33] Kenny PA, Lee GY, Myers CA, Neve RM, Semeiks JR (2007). The morphologies of breast cancer cell lines in three-dimensional assays correlate with their profiles of gene expression.. Mol Oncol.

[34] Regitnig P, Reiner A, Dinges HP, Hofler G, Muller-Holzner E (2002). Quality assurance for detection of estrogen and progesterone receptors by immunohistochemistry in Austrian pathology laboratories.. Virchows Arch.

[35] Guarneri V, Piacentini F, Ficarra G, Frassoldati A, D'Amico R (2009). A prognostic model based on nodal status and Ki-67 predicts the risk of recurrence and death in breast cancer patients with residual disease after preoperative chemotherapy.. Ann Oncol.

[36] Garcia S, Dales JP, Jacquemier J, Charafe-Jauffret E, Birnbaum D (2007). c-Met overexpression in inflammatory breast carcinomas: automated quantification on tissue microarrays.. Br J Cancer.

[37] Mulligan AM, Pinnaduwage D, Bull SB, O'Malley FP, Andrulis IL (2008). Prognostic effect of basal-like breast cancers is time dependent: evidence from tissue microarray studies on a lymph node-negative cohort.. Clin Cancer Res.

[38] Nocito A, Kononen J, Kallioniemi OP, Sauter G (2001). Tissue microarrays (TMAs) for high-throughput molecular pathology research.. Int J Cancer.

[39] Rosen DG, Huang X, Deavers MT, Malpica A, Silva EG (2004). Validation of tissue microarray technology in ovarian carcinoma.. Mod Pathol.

[40] Halushka MK, Cornish TC, Lu J, Selvin S, Selvin E (2009). Creation, validation, and quantitative analysis of protein expression in vascular tissue microarrays.. Cardiovasc Pathol.

[41] Rubin MA, Dunn R, Strawderman M, Pienta KJ (2002). Tissue microarray sampling strategy for prostate cancer biomarker analysis.. Am J Surg Pathol.

[42] Kononen J, Bubendorf L, Kallioniemi A, Barlund M, Schraml P (1998). Tissue microarrays for high-throughput molecular profiling of tumor specimens.. Nat Med.

[43] Hassan S, Ferrario C, Mamo A, Basik M (2008). Tissue microarrays: emerging standard for biomarker validation.. Curr Opin Biotechnol.

[44] Charpin C, Secq V, Giusiano S, Carpentier S, Andrac L (2009). A signature predictive of disease outcome in breast carcinomas, identified by quantitative immunocytochemical assays.. Int J Cancer.

[45] Netto GJ, Watkins DL, Williams JW, Colby TV, dePetris G (2006). Interobserver agreement in hepatitis C grading and staging and in the Banff grading schema for acute cellular rejection: the “hepatitis C 3” multi-institutional trial experience.. Arch Pathol Lab Med.

