# Supplementary material for: Breast Cancer Diagnosis Using a Microfluidic Multiplexed Immunohistochemistry Platform
Source: PLoS One. 2010 May 3;5(5):e10441. doi: 10.1371/journal.pone.0010441 (PMC2862720; doi:10.1371/journal.pone.0010441)
Supplement: Table S1 — Comparison of scores obtained via whole-section analysis (control) versus the MMIHC platform in human breast cancer tissues (n = 105). For ER and PR: negative (−); weak (+); intermediate (++); strong (+++). For HER2: score 0 (−); score 1 (+); score 2 (++); score 3 (+++). For Ki-67: ≤5% (−); 5%<x≤20% (+); 20%<x≤40% (++); >40% (+++). The unit of tumor size is centimeter. DCIS, ductal carcinoma in situ. (0.92 MB DOC) [file pone.0010441.s001.doc]

**Table S1.** **Comparison of scores obtained via whole-section analysis (control) *versus* the MMIHC platform in human breast cancer tissues (*n*=105).**

| **Case** | **Age** | **Tumor size** | **Nodes** | **ER** | | **HER2** | | | **PR** | | **Ki-67** | |
| --- | --- | --- | --- | --- | --- | --- | --- | --- | --- | --- | --- | --- |
|  |  |  |  | **Control** | **MMIHC** | **Control** | **MMIHC** | **Control** | | **MMIHC** | **Control** | **MMIHC** |
| 1 | 40 | 1.3 | 0 | +++ | +++ | − | − | +++ | | +++ | ++ | ++ |
| 2 | 45 | 2.4 | 0 | +++ | +++ | − | − | +++ | | +++ | ++ | ++ |
| 3 | 61 | 2.7 | 2 | +++ | +++ | − | − | +++ | | +++ | + | + |
| 4 | 72 | 1.3 | 0 | +++ | +++ | − | − | − | | − | − | − |
| 5 | 43 | 0.9 | 0 | +++ | +++ | − | + | +++ | | +++ | ++ | ++ |
| 6 | 47 | 0.7 | 0 | − | − | ++ | ++ | − | | − | ++ | ++ |
| 7 | 44 | 0.8 | 0 | +++ | +++ | − | − | ++ | | − | +++ | ++ |
| 8 | 56 | 2.5 | 3 | +++ | +++ | − | − | +++ | | +++ | − | − |
| 9 | 62 | 1.1 | 0 | +++ | +++ | − | − | +++ | | +++ | + | + |
| 10 | 39 | 1.4 | 0 | +++ | +++ | − | − | +++ | | +++ | ++ | ++ |
| 11 | 50 | 1.4 | 0 | +++ | +++ | + | + | +++ | | +++ | + | + |
| 12 | 64 | 4 | 0 | +++ | +++ | − | − | +++ | | +++ | − | − |
| 13 | 43 | 1.4 | 1 | +++ | +++ | − | + | +++ | | +++ | ++ | ++ |
| 14 | 59 | 0.8 | 0 | +++ | +++ | − | − | +++ | | +++ | + | + |
| 15 | 59 | 2.2 | 0 | +++ | +++ | − | − | +++ | | +++ | − | − |
| 16 | 70 | 1.3 | 0 | +++ | +++ | − | − | +++ | | +++ | ++ | ++ |
| 17 | 38 | 1.6 | 6 | +++ | +++ | − | + | +++ | | +++ | − | − |
| 18 | 54 | 0.9 | 0 | +++ | +++ | − | − | +++ | | ++ | − | − |
| 19 | 60 | 1.4 | 0 | ++ | ++ | + | + | +++ | | +++ | ++ | ++ |
| 20 | 50 | 2.5 | 0 | +++ | +++ | +++ | +++ | ++ | | + | ++ | +++ |
| 21 | 48 | 5.5 | 11 | +++ | +++ | ++ | ++ | ++ | | ++ | +++ | +++ |
| 22 | 63 | 0.9 | 1 | +++ | +++ | − | − | +++ | | +++ | ++ | ++ |
| 23 | 47 | DCIS | 0 | ++ | ++ | − | − | ++ | | ++ | − | − |
| 24 | 45 | 2.5 | 0 | +++ | +++ | − | − | +++ | | +++ | − | − |
| 25 | 49 | 1.8 | 0 | + | + | − | − | − | | − | +++ | +++ |
| 26 | 27 | 1.5 | 0 | +++ | +++ | − | ++ | +++ | | +++ | + | + |
| 27 | 49 | 3.5 | 0 | ++ | +++ | − | + | +++ | | +++ | ++ | ++ |
| 28 | 32 | 1.8 | 0 | +++ | +++ | + | +++ | +++ | | +++ | +++ | +++ |
| 29 | 64 | 1.8 | 0 | +++ | +++ | − | − | +++ | | +++ | − | − |
| 30 | 71 | 2.4 | 0 | +++ | +++ | +++ | +++ | + | | ++ | + | + |
| 31 | 41 | 10 | 10 | +++ | +++ | ++ | ++ | +++ | | +++ | − | − |
| 32 | 45 | 1.9 | 1 | +++ | +++ | ++ | ++ | +++ | | +++ | + | + |
| 33 | 45 | 2 | 0 | +++ | +++ | + | − | ++ | | ++ | − | − |
| 34 | 59 | 2 | 0 | +++ | +++ | ++ | ++ | +++ | | +++ | − | − |
| 35 | 61 | 1.8 | 0 | +++ | +++ | − | − | +++ | | +++ | − | − |
| 36 | 51 | 2.2 | 0 | +++ | +++ | − | − | + | | + | − | − |
| 37 | 67 | 2.2 | 0 | +++ | +++ | − | − | +++ | | +++ | + | − |
| 38 | 46 | 1.5 | 0 | +++ | +++ | − | − | +++ | | +++ | + | + |
| 39 | 38 | 2.5 | 0 | +++ | +++ | − | − | + | | +++ | + | + |
| 40 | 47 | 1.2 | 0 | +++ | +++ | − | − | +++ | | +++ | − | − |
| 41 | 48 | 2.5 | 4 | +++ | +++ | − | − | ++ | | ++ | +++ | +++ |
| 42 | 45 | 1.5 | 0 | +++ | +++ | − | + | +++ | | +++ | − | − |
| 43 | 37 | 2 | 1 | +++ | +++ | − | − | +++ | | +++ | + | + |
| 44 | 48 | 1.7 | 1 | +++ | +++ | − | − | +++ | | +++ | − | − |
| 45 | 36 | 1.8 | 2 | +++ | +++ | − | − | +++ | | +++ | + | + |
| 46 | 46 | 2 | 1 | +++ | +++ | ++ | ++ | +++ | | +++ | ++ | ++ |
| 47 | 44 | 2.5 | 20 | +++ | +++ | − | − | +++ | | +++ | − | − |
| 48 | 41 | 2.5 | 0 | +++ | +++ | − | + | ++ | | ++ | + | + |
| 49 | 41 | 3 | 0 | +++ | +++ | − | − | +++ | | +++ | + | − |
| 50 | 64 | 1.4 | 0 | +++ | +++ | − | − | +++ | | +++ | − | − |
| 51 | 57 | 1.3 | 0 | +++ | +++ | − | + | +++ | | +++ | + | + |
| 52 | 48 | 1.8 | 1 | +++ | +++ | +++ | +++ | ++ | | ++ | ++ | + |
| 53 | 42 | 2 | 0 | +++ | +++ | − | − | +++ | | +++ | − | − |
| 54 | 48 | 2.3 | 0 | +++ | +++ | − | + | +++ | | +++ | − | − |
| 55 | 42 | 3.5 | 0 | +++ | +++ | +++ | +++ | ++ | | ++ | + | + |
| 56 | 48 | 1.8 | 12 | +++ | +++ | − | − | − | | − | − | − |
| 57 | 32 | 1.8 | 0 | +++ | +++ | ++ | ++ | +++ | | +++ | − | − |
| 58 | 49 | 2 | 1 | +++ | +++ | − | − | + | | + | − | − |
| 59 | 63 | 1.5 | 0 | +++ | +++ | − | − | +++ | | ++ | − | − |
| 60 | 42 | 4 | 0 | ++ | ++ | − | − | +++ | | ++ | − | − |
| 61 | 49 | 2 | 0 | + | + | − | − | + | | + | ++ | ++ |
| 62 | 61 | 1.5 | 0 | +++ | +++ | − | − | +++ | | +++ | − | − |
| 63 | 43 | 1.5 | 0 | +++ | +++ | − | − | +++ | | +++ | − | − |
| 64 | 42 | 2.2 | 0 | +++ | +++ | − | − | ++ | | ++ | +++ | ++ |
| 65 | 62 | 3 | 0 | +++ | +++ | − | − | +++ | | +++ | − | − |
| 66 | 78 | 2.5 | 3 | ++ | ++ | − | − | +++ | | +++ | − | − |
| 67 | 42 | 4 | 2 | +++ | +++ | − | − | +++ | | +++ | +++ | +++ |
| 68 | 44 | 1.8 | 1 | +++ | +++ | − | − | +++ | | ++ | + | + |
| 69 | 37 | 1.8 | 0 | +++ | +++ | ++ | +++ | +++ | | +++ | + | + |
| 70 | 37 | 2.2 | 1 | +++ | +++ | − | − | +++ | | +++ | + | + |
| 71 | 45 | 1.5 | 0 | +++ | +++ | − | − | +++ | | +++ | − | − |
| 72 | 43 | 1.8 | 2 | +++ | +++ | − | − | +++ | | +++ | − | − |
| 73 | 48 | 2.2 | 1 | +++ | +++ | − | − | +++ | | +++ | + | + |
| 74 | 38 | 3.5 | 1 | +++ | +++ | − | − | +++ | | +++ | − | − |
| 75 | 42 | 2 | 0 | +++ | +++ | − | − | +++ | | +++ | − | − |
| 76 | 48 | 3.8 | 12 | +++ | +++ | − | + | +++ | | +++ | + | + |
| 77 | 41 | 1.8 | 0 | +++ | +++ | − | − | +++ | | +++ | − | − |
| 78 | 46 | 2.5 | 1 | +++ | +++ | +++ | +++ | +++ | | +++ | +++ | +++ |
| 79 | 44 | 1.2 | 0 | +++ | +++ | − | − | +++ | | +++ | − | − |
| 80 | 36 | 2.5 | 1 | +++ | +++ | − | − | + | | + | + | − |
| 81 | 40 | 1.6 | 0 | +++ | +++ | − | − | +++ | | +++ | − | − |
| 82 | 65 | 1.8 | 0 | +++ | +++ | − | − | +++ | | +++ | + | + |
| 83 | 47 | 1.7 | 0 | +++ | +++ | − | − | +++ | | +++ | − | − |
| 84 | 48 | 2 | 1 | +++ | +++ | − | − | +++ | | +++ | − | − |
| 85 | 44 | 2 | 0 | + | + | +++ | +++ | +++ | | +++ | ++ | + |
| 86 | 66 | 1.6 | 0 | +++ | +++ | − | − | ++ | | − | − | − |
| 87 | 46 | 2 | 2 | +++ | +++ | − | − | +++ | | +++ | − | − |
| 88 | 49 | 2.4 | 1 | +++ | +++ | − | − | +++ | | +++ | + | + |
| 89 | 47 | 2 | 9 | ++ | ++ | − | − | ++ | | ++ | +++ | +++ |
| 90 | 41 | 1.2 | 0 | +++ | +++ | − | − | +++ | | +++ | − | − |
| 91 | 31 | 4 | 10 | +++ | +++ | − | − | +++ | | +++ | + | + |
| 92 | 41 | 2.6 | 2 | +++ | +++ | − | − | +++ | | +++ | − | − |
| 93 | 48 | 2.5 | 1 | ++ | +++ | +++ | +++ | ++ | | ++ | +++ | +++ |
| 94 | 37 | 2.5 | 5 | +++ | +++ | +++ | +++ | +++ | | +++ | + | + |
| 95 | 46 | 5 | 8 | +++ | +++ | − | − | +++ | | +++ | + | + |
| 96 | 46 | 2.5 | 0 | +++ | +++ | − | + | +++ | | +++ | + | + |
| 97 | 50 | 3 | 15 | +++ | +++ | − | − | +++ | | +++ | + | + |
| 98 | 36 | 1.8 | 0 | +++ | +++ | − | − | ++ | | ++ | + | + |
| 99 | 35 | 2.5 | 1 | +++ | +++ | − | − | +++ | | +++ | + | + |
| 100 | 46 | 3 | 6 | +++ | +++ | + | ++ | +++ | | +++ | ++ | ++ |
| 101 | 32 | 3.5 | 0 | +++ | +++ | − | ++ | +++ | | +++ | − | − |
| 102 | 34 | 2 | 0 | − | − | +++ | +++ | − | | − | +++ | +++ |
| 103 | 43 | 2 | 1 | − | − | − | − | − | | + | ++ | ++ |
| 104 | 35 | 2.5 | 0 | +++ | +++ | − | − | − | | − | + | − |
| 105 | 43 | 1.5 | 0 | − | − | − | − | − | | − | +++ | +++ |

For ER and PR: negative (–); weak (+); intermediate (++); strong (+++). For HER2: score 0 (−); score 1 (+); score 2 (++); score 3 (+++). For Ki-67: ≤ 5% (–); 5% < *x* ≤ 20% (+); 20% < *x* ≤ 40% (++); > 40% (+++). The unit of tumor size is centimeter. DCIS, ductal carcinoma in situ.
