# Supplementary material for: Breast Cancer Diagnosis Using a Microfluidic Multiplexed Immunohistochemistry Platform
Source: PLoS One. 2010 May 3;5(5):e10441. doi: 10.1371/journal.pone.0010441 (PMC2862720; doi:10.1371/journal.pone.0010441)
Supplement: Table S2 — Statistical concordance table of cyclic permutation test, including KCC, χ2 test and p-value. Six cases were tested for reproducibility where four slides were made from the breast tumor of the same patient (total number of tissues: 24). The KCCs were 1.00 for ER, 1.00 for HER2, 1.00 for PR, and 0.96 for Ki-67, respectively. The cyclic permutation tests indicate that the MMIHC platform is reproducible in tissue sample and the sequence of the biomarkers does not affect score results significantly. (0.76 MB DOC) [file pone.0010441.s002.doc]

**Table S2.** **Statistical concordance table of cyclic permutation test, including KCC, χ^2^ test and *p*-value.**

|  | KCC | χ^2^ | *p*-value |
| --- | --- | --- | --- |
| ER | 1.00 | 20.0 | <0.005 |
| HER2 | 1.00 | 20.0 | <0.005 |
| PR | 1.00 | 20.0 | <0.005 |
| Ki-67 | 0.96 | 19.2 | <0.005 |

Six cases were tested for reproducibility where four slides were made from the breast tumor of the same patient (total number of tissues: 24). The KCCs were 1.00 for ER, 1.00 for HER2, 1.00 for PR, and 0.96 for Ki-67, respectively. The cyclic permutation tests indicate that the MMIHC platform is reproducible in tissue sample and the sequence of the biomarkers does not affect score results significantly.
