# Supplementary figures and images for: Breast Cancer Diagnosis Using a Microfluidic Multiplexed Immunohistochemistry Platform
Source: PLoS One. 2010 May 3;5(5):e10441. doi: 10.1371/journal.pone.0010441 (PMC2862720; doi:10.1371/journal.pone.0010441)

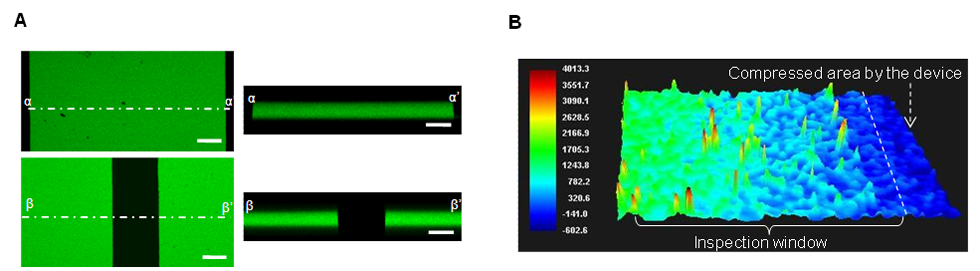

Supplement: Figure S1 — Characterization of the MMIHC platform. (A) Plane and z-stacked confocal laser microscopy images of the reaction channel area under 8 kPa. Reaction channels retained their original rectangular shape and each was separated completely. (B) Surface image of a cell block visualized using white light scanning interferometry. Cells in the reaction channels were intact under pressure, except for those in areas in direct contact with the MMIHC device. (0.25 MB TIF) [file pone.0010441.s003.tif]

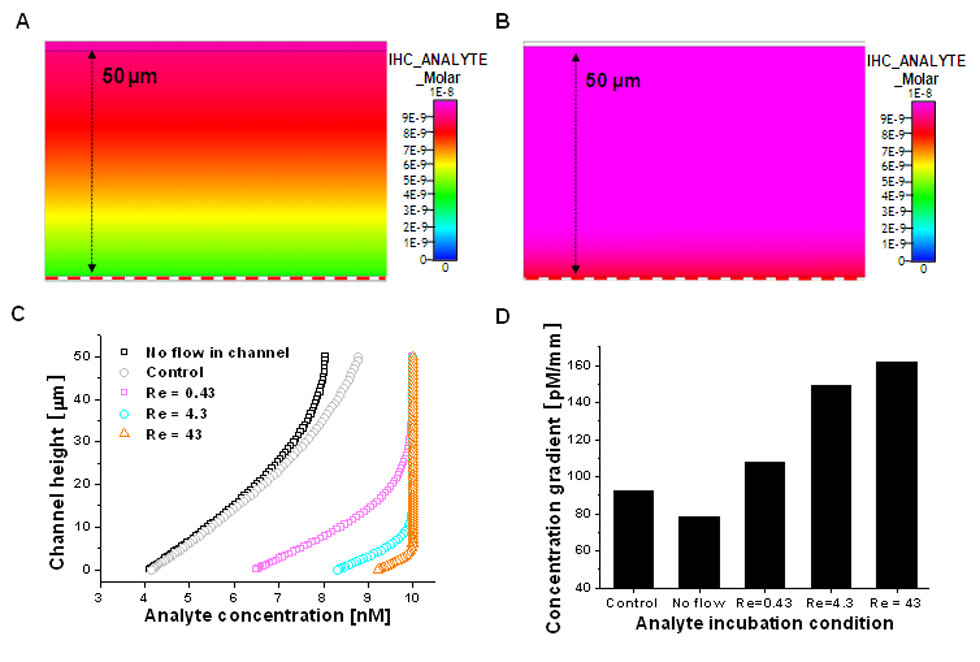

Supplement: Figure S2 — Computational fluid dynamics (CFD) study examining the kinetics of receptor-ligand binding. (A) Concentration profile of the analyte using the conventional method (transient state after 80 s). The red dotted line indicates a tissue sample with antigens. The analyte concentration in the vicinity of the tissue decreased with time because the tissue functioned as a sink. (B) Concentration profile of the analyte using the MMIHC platform (Re = 4.3; transient state after 80 s). Fresh analyte flowed into and was maintained in the vicinity of the tissue; therefore, the concentration of the analyte showed little decrease at the tissue surface as time progressed. (C) Concentration distribution of the analyte according to incubation conditions. The concentration profiles between a non-flowing microchannel and the conventional method were similar, and the concentration of the analyte exposed to the tissue was higher when the flow velocity of the analyte increased. (D) Concentration gradient versus analyte incubation conditions. When the flow velocity was high, diffusion of the analyte was dominant. (0.21 MB TIF) [file pone.0010441.s004.tif]

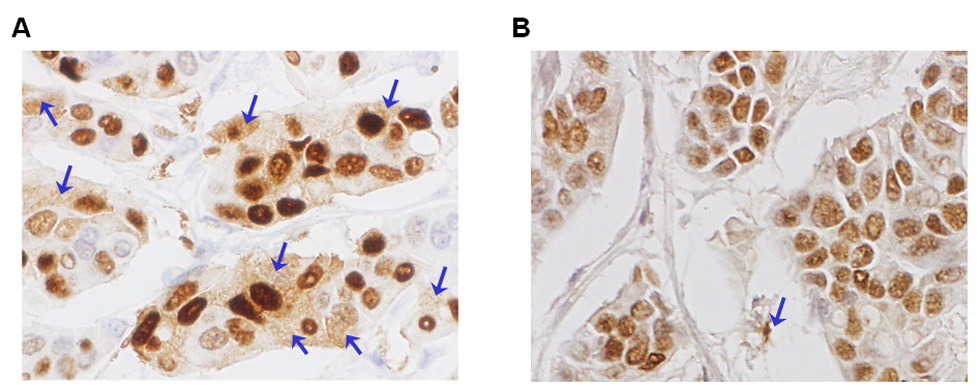

Supplement: Figure S3 — Images of immunohistochemical staining for PR. (A) An image of PR staining from automatic machine (×400). (B) An image of PR staining from the MMIHC platform (×400). Blue solid arrows indicate non-specific staining. Normally, more non-specific staining was shown in conventional automatic IHC machine. (0.68 MB TIF) [file pone.0010441.s005.tif]

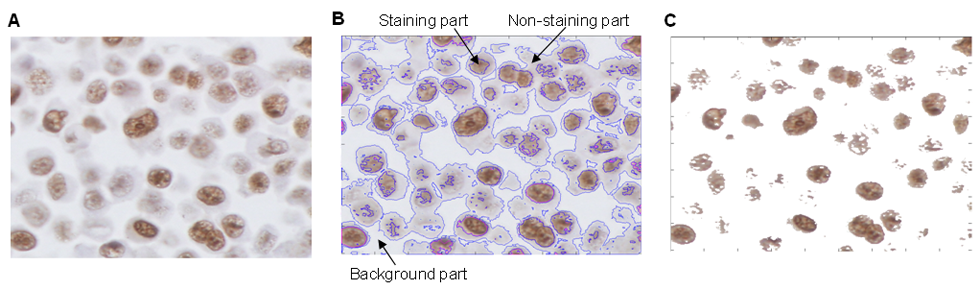

Supplement: Figure S4 — Image analysis of biomarker expression level. (A) A microscopic image acquired via MMIHC. (B) The image was divided into three parts: the staining part (SP), the non-staining part (NSP), and the background. Only the cell area (SP and NSP) was considered to minimize the variation of expression level according to cell density. (C) Image after analysis. Only the brown-colored areas remained. (0.36 MB TIF) [file pone.0010441.s006.tif]
